# Nanoparticle-mediated delivery of herbal-derived natural products to modulate immunosenescence-induced drug resistance in cancer therapy: a comprehensive review

**DOI:** 10.3389/fonc.2025.1567896

**Published:** 2025-04-28

**Authors:** Lichang Yang, Wei Wu, Jing Yang, Manman Xu

**Affiliations:** ^1^ Xuzhou Affiliated Hospital of Nanjing University of Chinese Medicine, Xuzhou, China; ^2^ Department of Geriatrics, Guang' anmen Hospital, China Academy of Chinese Medical Sciences, Beijing, China

**Keywords:** nanoparticles, herbal-derived natural products, immunosenescence, drug resistance, cancer therapy, tumor microenvironment

## Abstract

Immunosenescence, the age-associated decline of the immune system, is pivotal in fostering drug resistance within the tumor microenvironment (TME). The accumulation of senescent immune cells and the release of pro-inflammatory senescence-associated secretory phenotype (SASP) factors create a milieu that supports tumor survival and undermines therapeutic efficacy. Traditional cancer treatments often fail to address this underlying issue, leading to suboptimal outcomes. This article proposes an innovative strategy to overcome immunosenescence-induced drug resistance through the nanoparticle-mediated delivery of herbal-derived natural products (HDNPs), which possess senolytic and immunomodulatory properties capable of clearing senescent cells and rejuvenating immune function. Nanoparticle delivery systems enhance these compounds’ stability, bioavailability, and targeted delivery to the TME and senescent immune cells. By harnessing the synergistic effects of HDNPs and nanotechnology, this approach offers a novel and multifaceted solution to drug resistance in cancer therapy. It holds the potential to restore immune surveillance, reduce pro-survival signaling in cancer cells, and enhance the efficacy of conventional treatments. This paradigm shift emphasizes the importance of addressing immunosenescence as a therapeutic target and paves the way for more effective and personalized cancer interventions.

## 1 Introduction

Cancer remains a leading cause of mortality globally, with millions of new cases diagnosed each year. Despite significant advancements in oncology, the efficacy of cancer treatments is often hindered by the development of drug resistance, which is a major obstacle to successful therapy (1). Drug resistance can be intrinsic or acquired and involves complex mechanisms that allow cancer cells to evade the cytotoxic effects of chemotherapeutic agents (2). Key mechanisms of drug resistance include increased drug efflux via overexpression of transporter proteins, alterations in drug targets, activation of DNA repair pathways, evasion of apoptosis, and metabolic reprogramming (3). The tumor microenvironment (TME) also plays a crucial role by providing a protective niche that supports cancer cell survival and contributes to resistance (4). The heterogeneity of tumors adds another layer of complexity, as different cell populations within the same tumor may respond variably to treatment (5). This intratumoral diversity facilitates the selection of resistant clones under therapeutic pressure, leading to treatment failure and disease progression. Emerging evidence suggests that immunosenescence—the gradual deterioration of the immune system associated with aging—significantly contributes to cancer progression and drug resistance (6). Immunosenescence affects innate and adaptive immunity, leading to reduced immunosurveillance and an increased incidence of malignancies in older people (7). Within the TME, immunosenescent cells exhibit altered functionality, including decreased proliferation, impaired cytokine production, and reduced cytotoxic activity. These changes can create an immunosuppressive environment that fosters tumor growth and diminishes the efficacy of chemotherapy and immunotherapy. Moreover, senescent immune cells can secrete various pro-inflammatory cytokines, chemokines, and proteases collectively known as the senescence-associated secretory phenotype (SASP) (8). The SASP can promote tumor cell proliferation, angiogenesis, and metastasis while inhibiting apoptosis, thereby contributing to drug resistance. Understanding the interplay between immunosenescence and drug resistance is crucial for developing novel therapeutic strategies that can enhance treatment efficacy, particularly in aging populations where cancer incidence is highest (9).

Immunosenescence is characterized by accumulated aged immune cells that have reached replicative senescence due to telomere shortening or have become senescent in response to stress and DNA damage (10). These cells display altered surface markers, such as increased expression of inhibitory receptors (e.g., PD-1, CTLA-4) and decreased levels of costimulatory molecules. Functionally, they exhibit diminished proliferative capacity, reduced cytokine production, and impaired ability to eliminate tumor cells (11). In the TME, immunosenescent cells contribute to drug resistance through several mechanisms. Firstly, senescent immune cells secrete SASP factors that promote tumor cell survival and proliferation. Interleukin-6 (IL-6) and interleukin-8 (IL-8) can activate survival pathways in cancer cells, leading to resistance against apoptosis induced by chemotherapy (12). Secondly, the reduced cytotoxicity of senescent T cells and natural killer (NK) cells diminishes the immune system’s ability to recognize and eliminate cancer cells, allowing resistant clones to expand (13). Thirdly, immunosenescence is associated with an increase in regulatory T cells (Tregs) and myeloid-derived suppressor cells (MDSCs), which further suppress anti-tumor immune responses and contribute to a permissive environment for tumor growth (14). In addition, senescent immune cells can influence other components of the TME, such as fibroblasts and endothelial cells, enhancing angiogenesis and extracellular matrix remodeling that facilitate tumor progression and metastasis (15). These factors collectively create a TME that supports cancer cell survival and proliferation while undermining the effectiveness of therapeutic agents.

Herbal-derived natural products (HDNPs) have garnered significant interest due to their diverse bioactive compounds with immunomodulatory, anti-inflammatory, and anti-cancer properties. Compounds such as quercetin, fisetin, and epigallocatechin gallate (EGCG) have demonstrated the ability to modulate immune cell function, reduce SASP factor secretion, and induce apoptosis in senescent cells (16). These HDNPs can target multiple signaling pathways involved in immunosenescence and drug resistance. Quercetin has been shown to inhibit the NF-κB pathway, reducing the production of pro-inflammatory cytokines (17). Fisetin can activate the Nrf2 pathway, enhancing the antioxidant capacity of cells and reducing oxidative stress (18). EGCG has been reported to modulate epigenetic modifications, restoring the function of aging immune cells (19). Despite their therapeutic potential, HDNPs often face challenges such as poor solubility, low bioavailability, and rapid metabolism, which limit their clinical application. Nanoparticle-based delivery systems offer a promising solution to these issues by enhancing the stability and bioavailability of HDNPs (20). Nanoparticles can be engineered to deliver HDNPs specifically to the TME and senescent immune cells, minimizing systemic toxicity and improving therapeutic efficacy. Features such as controlled release, protection from degradation, and surface modification with targeting ligands enable precise delivery and sustained therapeutic action (21). Moreover, nanoparticles can cross biological barriers and accumulate in tumors via the enhanced permeability and retention (EPR) effect, further increasing the concentration of HDNPs at the desired site (21). This targeted approach enhances the anti-cancer effects of HDNPs and allows for the modulation of immunosenescence within the TME, potentially reversing drug resistance mechanisms.

This article explores the innovative approach of utilizing nanoparticles to deliver HDNPs that modulate immunosenescence-induced drug resistance in cancer therapy. By integrating insights from oncology, immunology, and nanotechnology, we seek to comprehensively analyze how nanoparticle-mediated HDNP delivery can rejuvenate aging immune cells, suppress SASP factors, and enhance anti-tumor immunity. This strategy holds promise for overcoming drug resistance and improving therapeutic outcomes in cancer patients, particularly the older.

## 2 Immunosenescence-induced drug resistance in cancer

Immunosenescence refers to the gradual deterioration of the immune system associated with aging, leading to an increased susceptibility to infections, diseases, and cancer progression (22). Within the TME, immunosenescence is characterized by the accumulation of senescent immune cells, including T cells, NK cells, and dendritic cells (DCs). These senescent cells exhibit altered phenotypes and diminished functional capacities, such as reduced proliferative ability and impaired cytokine production. The accumulation of senescent immune cells is driven by persistent antigen exposure, telomere shortening, and oxidative stress (23). Repetitive stimulation of T cells by tumor antigens can induce a senescent state, which in turn upregulates the expression of inhibitory receptors like PD-1 and TIM-3, leading to T cell exhaustion (24). This immunosenescent profile contributes to a diminished immune response against tumor cells. Furthermore, senescent immune cells secrete various pro-inflammatory cytokines, chemokines, growth factors, and proteases collectively known as SASP (25). The SASP can have paradoxical effects within the TME. While initially intended to recruit immune cells for clearance of senescent cells, the chronic presence of SASP factors can promote tumorigenesis (25). SASP components such as IL-6, IL-8, and vascular endothelial growth factor (VEGF) enhance tumor cell proliferation, angiogenesis, and metastasis (25). Additionally, matrix metalloproteinases (MMPs) secreted by senescent cells degrade the extracellular matrix, facilitating cancer cell invasion (26). The SASP thus creates a pro-tumorigenic environment that supports cancer progression. The altered cytokine milieu from immunosenescence fosters an environment conducive to drug resistance. Pro-inflammatory cytokines like IL-6 and tumor necrosis factor-alpha (TNF-α) activate survival signaling pathways in cancer cells, such as the STAT3 and NF-κB pathways (27). Activation of these pathways upregulates anti-apoptotic proteins (Bcl-2, Bcl-xL) and drug efflux transporters (P-glycoprotein), reducing the efficacy of chemotherapeutic agents (28). Furthermore, SASP factors can induce epithelial-to-mesenchymal transition (EMT) in cancer cells, associated with increased resistance to chemotherapy and targeted therapies (29). EMT endows cancer cells with stem cell-like properties, enhancing their survival ability in the presence of anticancer drugs.

On the other hand, immunosenescence impairs the immune system’s ability to recognize and eliminate cancer cells, contributing to immune evasion and therapeutic resistance (30). Senescent T cells exhibit decreased cytotoxic activity and reduced interferon-gamma (IFN-γ) production, a critical cytokine for anti-tumor immunity. Similarly, senescent NK cells show diminished expression of activating receptors and impaired degranulation capacity (30). This compromised immune surveillance allows tumor cells to proliferate unchecked and develop mechanisms to resist therapy. Reduced immune pressure can enable the expansion of cancer cell clones with mutations conferring drug resistance. Additionally, the immunosuppressive environment can inhibit the effectiveness of immunotherapies designed to reactivate immune responses against tumors (31). Recent research has highlighted the direct involvement of immunosenescent cells in promoting drug resistance. A study demonstrated that senescent CD8^+^ T cells in the TME secrete IL-10, suppressing the effector T cells’ function and contributing to resistance against anti-PD-1 therapy in melanoma models (32). The depletion of senescent T cells restored responsiveness to immunotherapy, indicating their role in therapeutic failure. Another study by Di Mitri et al. showed that senescent stromal cells in prostate cancer release SASP factors that activate the IL-6/STAT3 pathway in tumor cells, leading to resistance to chemotherapy (33). Blocking IL-6 signaling sensitized cancer cells to treatment, further establishing the link between immunosenescence and drug resistance.

**Figure 1:**
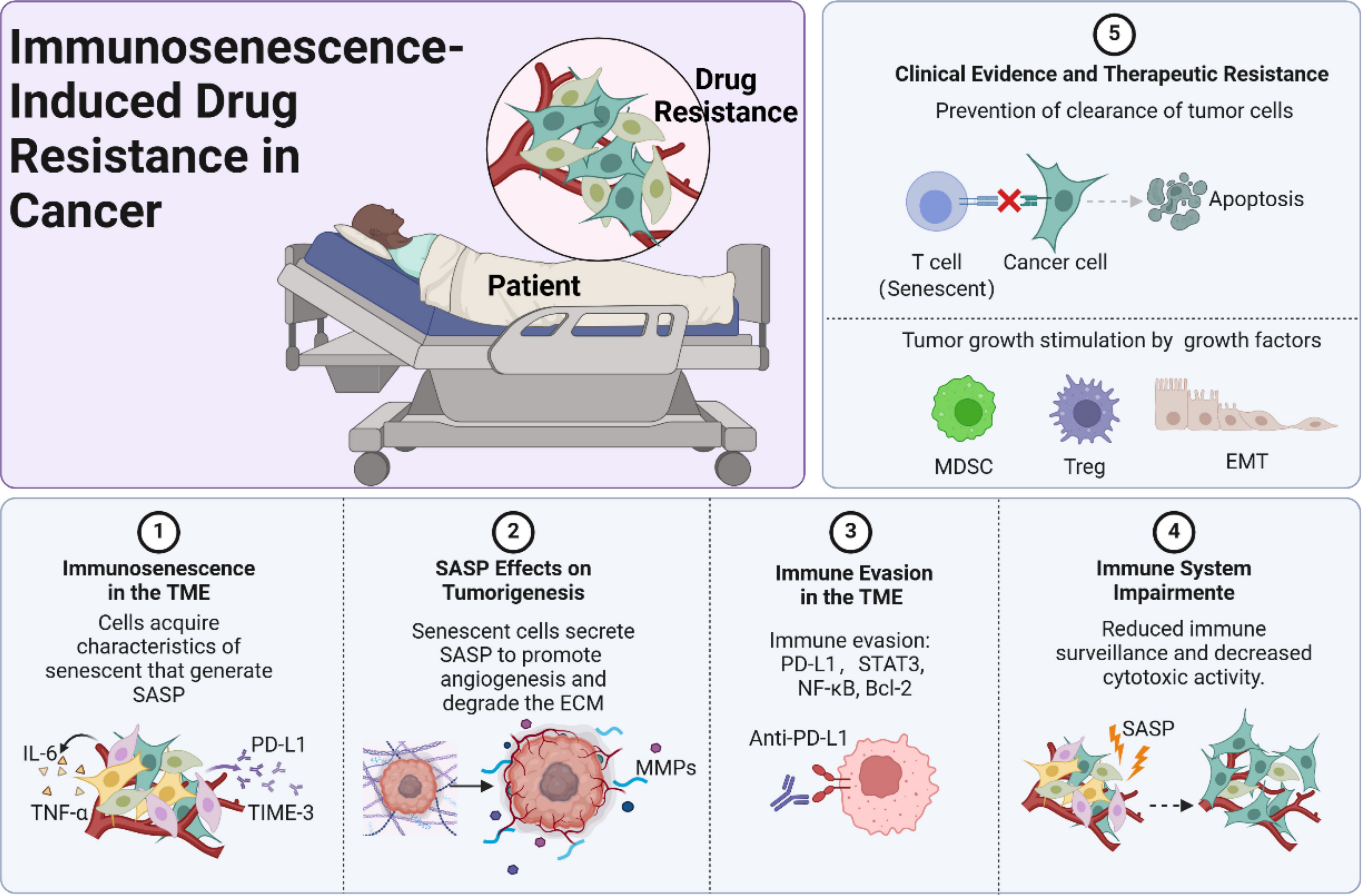
Immunosenescence-induced drug resistance in cancer.

Clinical data also support the association between immunosenescence and poor treatment outcomes in cancer patients. Older patients often exhibit reduced responses to chemotherapy and immunotherapy compared to younger individuals (34). This disparity is partly attributed to age-related declines in immune function, including decreased T cell diversity and function. A retrospective analysis by Elias et al. found that older patients with non-small cell lung cancer had lower overall survival rates following immunotherapy, correlating with markers of immunosenescence (35). Similarly, studies have shown that high levels of senescent immune cells in peripheral blood are associated with decreased progression-free survival in patients undergoing targeted therapies (36). These findings underscore the impact of immunosenescence on therapeutic efficacy and highlight the need for interventions that address immune aging to overcome drug resistance (**Figure 1**).

## 3 Herbal-derived natural products targeting immunosenescence

### 3.1 HDNPs with anti-immunosenescence properties

**Figure 2:**
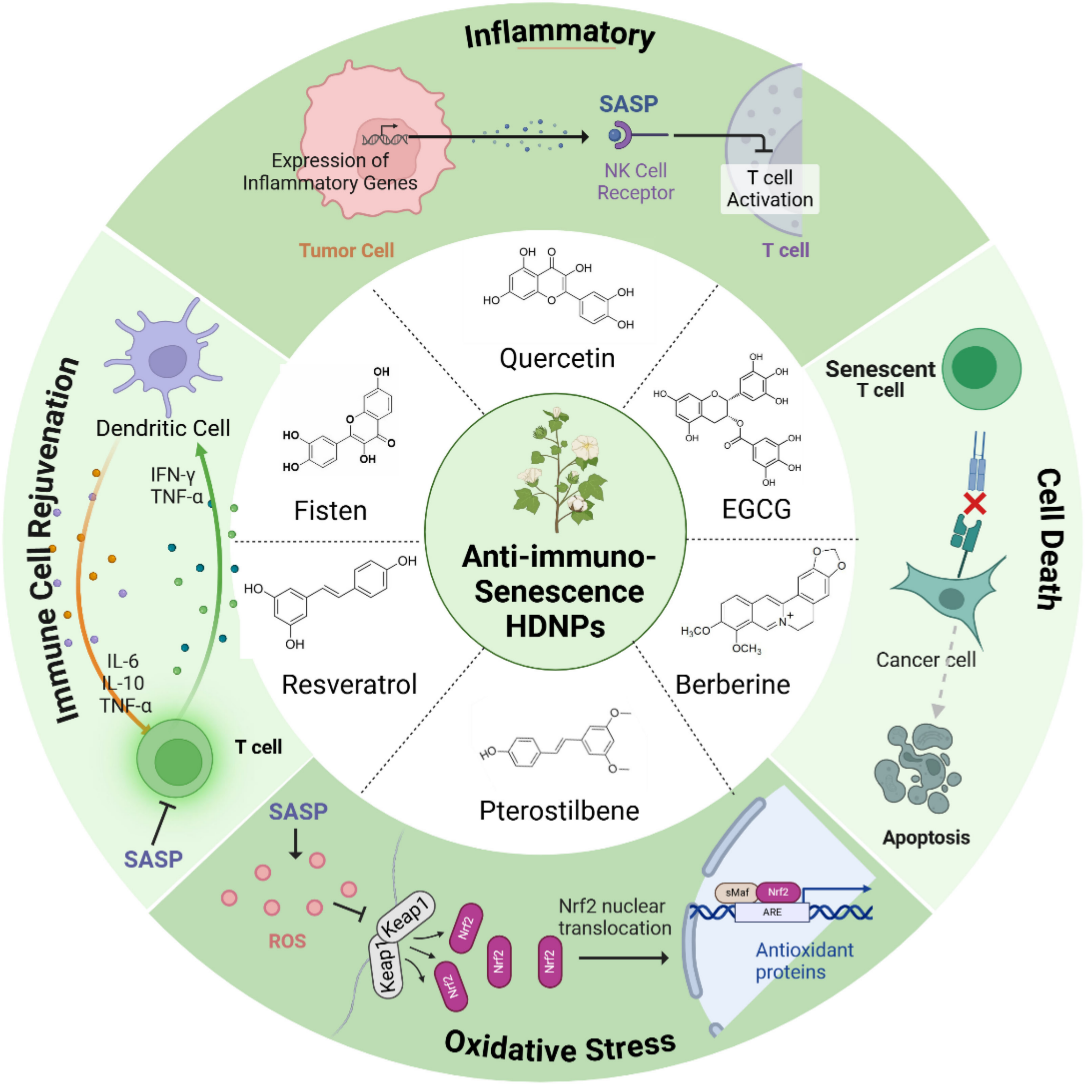
HDNPs with anti-immunosenescence properties and their mechanisms of action.

HDNPs possess significant potential to mitigate immunosenescence and reverse drug resistance in cancer therapy. Compounds such as quercetin, fisetin, EGCG, resveratrol, curcumin, and others have demonstrated unique capabilities to modulate immune aging mechanisms within the TME (**Figure 2**).

Quercetin, abundant in fruits and vegetables, selectively induces apoptosis in senescent cells by targeting anti-apoptotic pathways unique to these cells, thus reducing the tumor-supportive milieu created by senescent cells (37, 38). Fisetin, primarily found in strawberries and apples, effectively decreases the secretion of SASP factors such as IL-6 and IL-8, reducing inflammation and rejuvenating the proliferative capacity of cytotoxic immune cells including T cells and NK cells (39, 40). EGCG from green tea modulates immune signaling pathways via suppression of NF-κB and AP-1, thus attenuating pro-inflammatory gene expression and fostering immune homeostasis through Treg cell modulation and anti-inflammatory differentiation of naïve T cells (41, 42). Resveratrol, found in grapes and berries, enhances immune function and delays immune cell senescence by activating longevity-associated pathways such as SIRT1 and AMPK, thereby reinforcing antitumor immune surveillance (43). Curcumin, derived from turmeric, exhibits anti-inflammatory properties by suppressing NF-κB signaling, reducing SASP-related inflammation (44). Ginsenosides from *Panax ginseng* demonstrate immunomodulatory effects that combat immune aging, potentially rejuvenating T-cell and NK-cell functions (45). Similarly, Astragaloside IV promotes telomerase activity, delaying cellular senescence and enhancing immune cell functionality (46). Berberine suppresses pro-inflammatory SASP factors through inhibition of the NF-κB pathway, while sulforaphane induces apoptosis specifically in senescent cells, reducing their accumulation (47, 48). Genistein, an isoflavone in soy products, further contributes by inhibiting inflammatory signaling pathways critical in SASP regulation (49) (**Table 1**).

**Table 1:** Mechanistic insights into the effects of HDNPs on immunosenescence in the TME.

HDNP	Primary Source	Anti-Immunosenescence Effects	Mechanisms of Action	References
Quercetin	Apples, onions, berries	Selective apoptosis of senescent cells; reduction of SASP	Inhibition of anti-apoptotic proteins (Bcl-xL, eEF1A), suppression of NF-κB pathway, enhancing CTLs and NK cells function	(37, 38, 50, 51, 54)
Fisetin	Strawberries, apples, persimmons	Reduction of SASP factors, rejuvenation of immune cells	mTOR inhibition, SIRT1 activation, enhancement of T cell and NK cell proliferation and function, improved antigen presentation by dendritic cells	(39, 40, 52, 55)
Epigallocatechin gallate (EGCG)	Green tea	Reduction of pro-inflammatory gene expression; balanced immune response	NF-κB and AP-1 pathway inhibition, enhancement of Treg function, promotion of anti-inflammatory T cell differentiation	(41, 42, 53)
Resveratrol	Grapes, berries, peanuts	Delay in immune cell senescence; enhanced immune surveillance	Activation of SIRT1 and AMPK pathways, delayed T cell senescence, improved proliferative capacity	(43)
Curcumin	Turmeric (Curcuma longa)	Anti-inflammatory; reduction of SASP	Inhibition of NF-κB signaling, reduced inflammatory cytokine secretion	(44)
Ginsenosides	Ginseng (Panax ginseng)	Immunomodulation; rejuvenation of T and NK cells	Modulation of immune aging processes	(45)
Astragaloside IV	Astragalus membranaceus	Delay in cellular senescence; enhanced immune function	Promotion of telomerase activity, improved T cell and NK cell functions	(46)
Berberine	Berberis vulgaris	Reduction of SASP factors; anti-inflammatory	Suppression of NF-κB pathway, decreased cytokine production	(47)
Sulforaphane	Cruciferous vegetables (e.g., broccoli)	Selective apoptosis of senescent cells	Induction of apoptosis specifically in senescent cells	(48)
Genistein	Soy products	Reduction of inflammatory signaling	Inhibition of pro-inflammatory signaling pathways critical to SASP regulation	(49)
Luteolin (in Salvia haenkei - Haenkenium)	Salvia haenkei	Prevention of cellular senescence; reduction of cardiotoxicity induced by chemotherapy	Disruption of p16–CDK6 interaction, suppression of senescence phenotypes	(56, 57)
Pterostilbene	Blueberries, grapes	Anti-aging; enhancement of cellular health and longevity	Modulation of sirtuin pathways, increased NAD+ levels, activation of Nrf2/Keap1 antioxidant pathway	(57)

### 3.2 Mechanisms of action

HDNPs counter immunosenescence through three primary mechanisms: senolytic activity, inflammation modulation, and immune cell rejuvenation.

As senolytics, quercetin and fisetin selectively induce apoptosis in senescent cells via inhibition of anti-apoptotic proteins such as Bcl-xL and eEF1A, significantly reducing SASP burden and tumor-promoting inflammation (50, 51). Fisetin’s ability to modulate mTOR and activate SIRT1 pathways further reduces secretion of pro-inflammatory cytokines IL-6 and IL-8, thus limiting inflammatory signaling in the TME (52). EGCG similarly decreases NF-κB activation, curtailing chronic inflammation and hampering tumor survival pathways (53). In immune rejuvenation, quercetin enhances cytotoxic functions of CTLs and NK cells, significantly improving their tumor-eliminating capacity (54). Fisetin boosts antigen presentation capabilities by increasing co-stimulatory molecules on dendritic cells, facilitating enhanced T cell activation (55). EGCG promotes balanced immune responses by expanding Treg populations that mitigate excessive inflammation without compromising antitumor immunity (41). Additionally, Zumerle et al. demonstrated that a polyphenol-rich natural extract from *Salvia haenkei* (Haenkenium, HK) can delay aging in mice by lowering systemic senescence markers such as p16 and p27 (56). Luteolin in HK disrupts the p16–CDK6 interaction, preventing cell cycle arrest, suppressing senescence phenotypes, and mitigating doxorubicin-induced senescence and cardiotoxicity—thereby highlighting HK’s potential to reduce side effects and confer anti-aging benefits in cancer therapy (57). Additionally, pterostilbene exerts anti-aging and anti-senescent effects by modulating sirtuin pathways and enhancing NAD+ levels. This potent antioxidant—chemically similar to resveratrol but more bioavailable—can boost NAD+ up to 90% in 30 days when combined with nicotinamide riboside (NR), underscoring its promise for improving cellular health and longevity (57). Moreover, pterostilbene activates the Nrf2/Keap1 pathway, fortifying the cell’s antioxidant defenses and thereby improving resilience to oxidative stress—hallmarks of aging-related cellular deterioration (57) (**Figure 2**).

Despite these promising biological effects, HDNPs face substantial pharmacokinetic barriers including poor bioavailability, rapid systemic clearance, and potential off-target toxicity at high concentrations. To overcome these limitations, nanoparticle-based delivery systems offer significant advantages by enhancing stability, bioavailability, and targeted release of HDNPs to senescent cells within the TME. Functionalized nanoparticles provide precision delivery, improving therapeutic efficacy while minimizing systemic toxicity and adverse effects (20, 58–61) (**Table 1**).

## 4 Nanoparticle-mediated delivery of HDNPs

### 4.1 Improved stability and solubility

**Figure 3:**
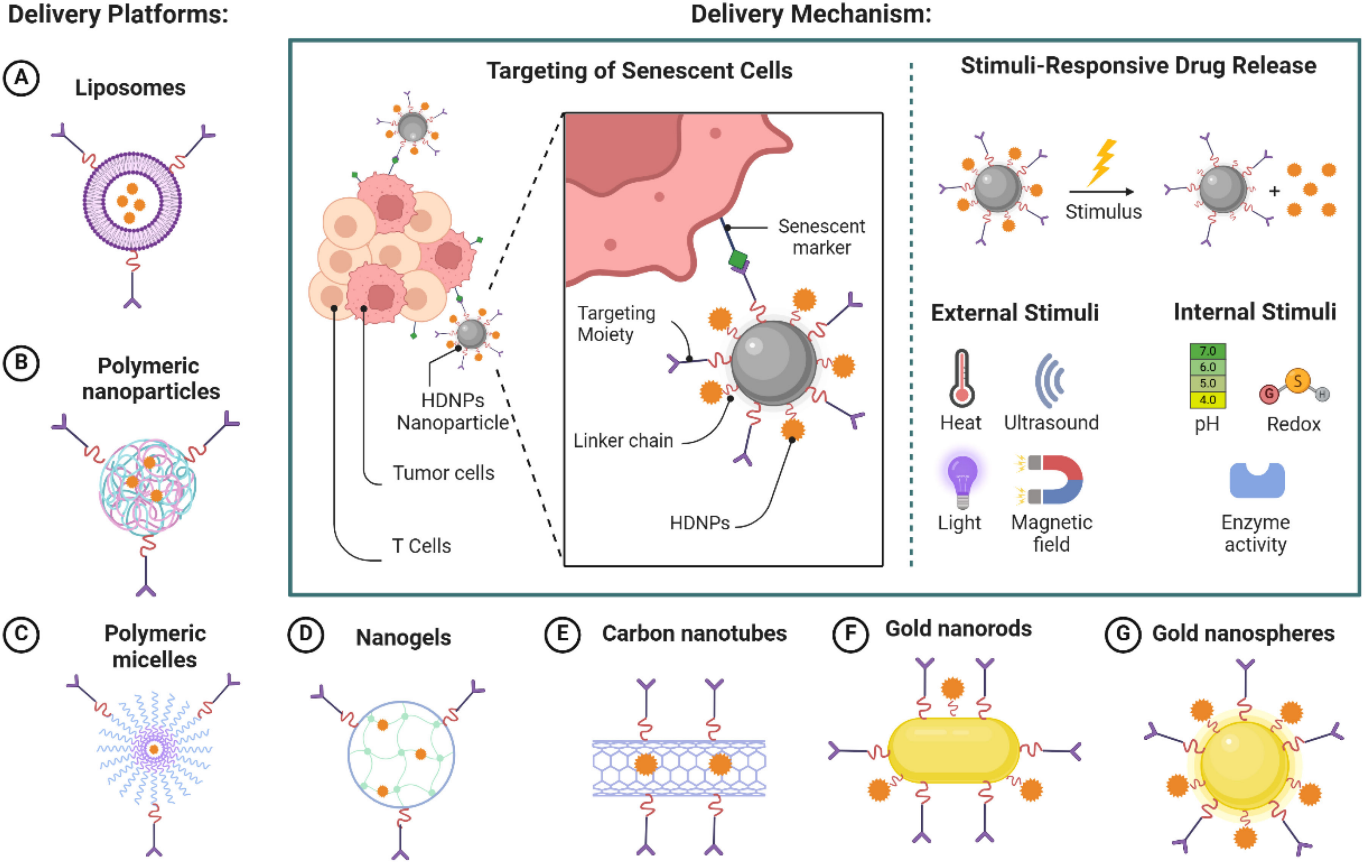
Nanoparticle-mediated delivery of HDNPs targeting senescent cells.

Incorporating HDNPs into nanoparticle delivery systems offers several advantages that enhance their therapeutic potential against immunosenescence-induced drug resistance in cancer therapy. Many HDNPs, such as quercetin and fisetin, possess poor water solubility and are unstable under physiological conditions, which limits their bioavailability and therapeutic efficacy. Encapsulating these compounds in nanoparticles, such as liposomes, polymeric nanoparticles, polymeric micelles, nanogels, carbon nanotubes, and gold nanoparticles, can significantly enhance their solubility and protect them from degradation (**Figure 3**). Nanoparticles can shield HDNPs from enzymatic metabolism and chemical hydrolysis, prolonging their systemic circulation time and increasing the likelihood of reaching target sites (20).

Nanoparticles can be engineered for targeted delivery to the TME and senescent immune cells. Selective accumulation at the desired site is achieved by modifying the surface of nanoparticles with specific ligands or antibodies that recognize senescence-associated markers. Although senescence-associated β-galactosidase (SA-β-gal) is commonly used, it lacks specificity due to its association with general lysosomal activity. Therefore, more robust targeting strategies integrate multiple markers—such as p16^INK4A^, p21^Cip1^, γ-H2AX, and SASP-related surface proteins—to more accurately identify senescent cells. This targeted approach enhances the local concentration of HDNPs, maximizing therapeutic effects while minimizing off-target toxicity.

### 4.2 Controlled release profiles

Nanoparticle delivery systems can be designed to release controlled HDNPs that respond to specific stimuli within the TME. Controlled release mechanisms, such as pH-sensitive or enzyme-responsive systems, ensure that HDNPs are released at the site of action over an extended period (62). This maintains effective therapeutic concentrations and reduces systemic exposure and side effects, improving patient compliance and treatment outcomes. To effectively modulate immunosenescence, nanoparticles must be strategically designed to deliver HDNPs specifically to senescent immune cells within the TME. Senescent cells express unique biomarkers that can be exploited for targeted drug delivery (63). Nanoparticles can be functionalized with ligands or antibodies that specifically bind to the markers, enhancing uptake by senescent cells (64). This targeted delivery ensures that HDNPs exert their senolytic effects primarily on senescent cells, reducing the burden of the senescent cell population within the TME.

The TME exhibits distinct characteristics, such as acidic pH, hypoxia, and elevated levels of certain enzymes, which can be utilized to trigger the release of HDNPs from nanoparticles (65). Designing stimuli-responsive nanoparticles allows for: I) PH-sensitive release: Nanoparticles remain stable at physiological pH but release their payload in the acidic environment of the TME (pH ~6.5) or within endosomal/lysosomal compartments (pH ~5.0) after cellular uptake (66). II) Enzyme-responsive release: Nanoparticles degrade and release HDNPs in the presence of enzymes overexpressed in the TME, such as MMPs (67). These design strategies ensure that HDNPs are released precisely where needed, enhancing efficacy and reducing systemic toxicity (**Figure 3**).

### 4.3 Examples of nanoparticle-HDNP systems

Liposomes are versatile nanocarriers composed of phospholipid bilayers capable of encapsulating hydrophilic and hydrophobic compounds. Quercetin-loaded liposomes have been developed to target senescent fibroblasts within the TME (68). Recent studies have demonstrated that liposomes can be engineered with different surface properties, such as cationic, anionic, and zwitterionic characteristics, to optimize their interaction with cellular membranes and improve their therapeutic efficacy (69). Research indicates that quercetin and rutin, when encapsulated in cationic liposomes, can inhibit the toxic effects of empty liposomes, thereby enhancing cell viability (69). This targeted approach aims to deliver quercetin more effectively and exploit the unique properties of the TME, where senescent cells often reside, to improve therapeutic outcomes in cancer treatment. Resveratrol-loaded nanoparticles have been engineered to enhance targeting and uptake in immunosenescent cells within the TME, specifically by modulating nanoparticle surface chemistry and functional groups to bind receptors upregulated in senescent cells (70). Mechanistically, these nanoparticles utilize ligand-mediated binding to specific markers such as SA-β-gal or CD38, which are highly expressed in immunosenescent cells. While SA-β-gal is commonly employed, CD38 is increasingly recognized as an immunosenescence marker due to its elevated expression in aged immune cells, where it contributes to NAD+ degradation, cellular metabolic dysregulation, and impaired immune function (71). Once internalized, the resveratrol payload exerts senolytic and senostatic effects, inhibiting pro-inflammatory cytokine release (SASP factors) and restoring immune functionality through SIRT1 activation, downregulation of NF-κB signaling, and attenuation of oxidative stress (70). This approach selectively targets immunosenescent cells and reduces chronic inflammation in the TME, potentially enhancing immune cell infiltration and improving therapeutic responses to cancer immunotherapy (**Table 2**)​.

**Table 2:** Summary of nanoparticle-HDNP systems in cncer therapy.

HDNP	Nanoparticle System	Composition	Targeted Delivery Strategy	Therapeutic Outcome	References
Quercetin	Liposomes	Phospholipid bilayer encapsulating quercetin	Surface modification with ligands targeting senescent fibroblasts in TME	Enhanced delivery to senescent cells; improved therapeutic efficacy	(68)
Rutin	Cationic Liposomes	Positively charged liposomes with rutin	Exploiting electrostatic interactions with negatively charged cell membranes	Increased cell viability; reduced toxicity of empty liposomes	(69)
Resveratrol	Functionalized Nanoparticles	Nanoparticles with surface chemistry tailored for resveratrol	Ligand-mediated binding to SA-β-gal and CD38 markers on immunosenescent cells	Selective targeting; modulation of SASP; restored immune function	(70)
Curcumin	Polymeric Nanoparticles	Biodegradable polymers encapsulating curcumin	Passive targeting via enhanced permeability and retention (EPR) effect	Improved bioavailability; enhanced tumor suppression	(108)
Epigallocatechin Gallate (EGCG)	Gold Nanoparticles	EGCG-conjugated gold nanoparticles	Targeting through enhanced retention in tumor tissues	Induced apoptosis in cancer cells; reduced tumor growth	(109)
Berberine	Solid Lipid Nanoparticles	Lipid-based nanoparticles containing berberine	Passive targeting via EPR effect	Enhanced cytotoxicity against cancer cells; improved pharmacokinetics	(110)
Genistein	Polymeric Micelles	Amphiphilic copolymers forming micelles with genistein	Targeting estrogen receptor-positive cancer cells	Increased solubility; enhanced anti-tumor activity	(111)
Paclitaxel	Folate-Modified Pullulan Nanoparticles	Conjugated with folic acid and loaded with paclitaxel	Folate receptor-mediated endocytosis in cancer cells	Targeted delivery; reduced toxicity; improved therapeutic index	(112)
Camptothecin	Dendrimer Nanoparticles	PAMAM dendrimers encapsulating camptothecin	Surface modification for targeted delivery to tumor cells	Increased solubility; improved anti-cancer efficacy	(113)
10-Hydroxycamptothecin	Polymeric Micelles	Self-assembled pullulan micelles with 10-hydroxycamptothecin	Passive targeting via EPR effect	Improved stability; enhanced anti-tumor activity	(114)
Docetaxel	Polymeric Nanoparticles	Biodegradable polymer nanoparticles encapsulating docetaxel	Passive targeting via EPR effect	Enhanced bioavailability; improved therapeutic outcomes	(115)
Anthocyanins	Lipid-Based Nanoparticles	Lipid nanoparticles containing anthocyanins	Passive targeting via EPR effect	Enhanced stability; improved anti-cancer efficacy	(116)
Ellagic Acid	Polymeric Nanoparticles	Polymeric nanoparticles encapsulating ellagic acid	Passive targeting via EPR effect	Increased bioavailability; enhanced tumor suppression	(117)
Silibinin	Solid Lipid Nanoparticles	Lipid-based nanoparticles containing silibinin	Passive targeting via EPR effect	Improved solubility; enhanced anti-tumor activity	(118)
Baicalein	Polymeric Micelles	Amphiphilic copolymers forming micelles with baicalein	Passive targeting via EPR effect	Increased solubility; improved therapeutic efficacy	(119)
Betulinic Acid	Liposomes	Phospholipid bilayer encapsulating betulinic acid	Passive targeting via EPR effect	Enhanced bioavailability; improved anti-cancer activity	(120)
Emodin	Magnetic Nanoparticles	Emodin-conjugated PEGylation of Fe3O4 nanoparticles	Targeting through enhanced retention in tumor tissues	Induced apoptosis; reduced tumor growth	(121)
Gambogic Acid	Polymeric Nanoparticles	Biodegradable polymers encapsulating gambogic acid	Passive targeting via EPR effect	Enhanced bioavailability; improved anti-tumor efficacy	(122)
Honokiol	Solid Lipid Nanoparticles	Lipid-based nanoparticles containing honokiol	Passive targeting via EPR effect	Increased solubility; enhanced therapeutic outcomes	(99)
Isoliquiritigenin	Polymeric Prodrug Micelles	Isoliquiritigenin-loaded platinum (IV) prodrug micelles	Passive targeting via EPR effect	Improved stability; enhanced anti-cancer activity	(123)
Luteolin	Liposomes	Phospholipid bilayer encapsulating luteolin	Passive targeting via EPR effect	Enhanced bioavailability; improved tumor suppression	(56, 57)
Magnolol	Nanomicelles	Adenosine monophosphate modified nanomicelles containing magnolol	Targeting through enhanced retention in tumor tissues	Induced apoptosis; reduced tumor growth	(124)
Naringenin	Polymeric Nanoparticles	Polymeric nanoparticles encapsulating naringenin	Passive targeting via EPR effect	Increased bioavailability; enhanced anti-tumor efficacy	(125)
Oridonin	Solid Lipid Nanoparticles	PEG-PLGA-based nanoparticles with oridonin	Passive targeting via EPR effect	Increased bioavailability; enhanced anti-tumor efficacy	(126)

Not only that but innovative nanoparticle designs for HDNP delivery are also needed. For example, the development of nanoparticles that respond to multiple TME stimuli (e.g., pH, redox conditions) to achieve precise spatiotemporal release of HDNPs (72). Besides, incorporating imaging agents into HDNP-loaded nanoparticles for theranostic applications allows simultaneous cancer therapy and monitoring (73). Meanwhile, the combination therapies are also important (74). Designing nanoparticles co-loaded with HDNPs and chemotherapeutic agents to achieve synergistic effects in overcoming drug resistance. Nanoparticles loaded with HDNPs offers a promising strategy to modulate immunosenescence-induced drug resistance in cancer therapy. By improving the pharmacokinetic profiles of HDNPs and enabling targeted delivery to senescent cells within the TME, nanoparticles enhance therapeutic efficacy while minimizing systemic side effects (**Table 2**). Beyond their application in HDNP-based therapies, nanoparticle-mediated delivery systems represent a broadly applicable platform for enhancing the pharmacological performance of diverse therapeutic agents. Their versatility, biocompatibility, and capacity for targeted delivery make them attractive not only for cancer treatment but also for addressing a wide range of diseases globally. These advantages highlight the potential of nanoparticle systems to serve as a foundational strategy in the development of next-generation therapeutics.

## 5 Overcoming drug resistance by targeting immunosenescence with HDNP-loaded nanoparticles

### 5.1 Modulation of pro-survival signaling and SASP factors

Immunosenescence—the gradual decline of the immune system due to aging—plays a pivotal role in fostering an immunosuppressive TME, which significantly contributes to cancer progression and the development of drug resistance. This process involves the accumulation of senescent immune cells that secrete the SASP. The SASP not only promotes tumor growth but also enhances the survival and proliferation of cancer cells, thereby impeding the efficacy of various therapeutic interventions (15).

HDNPs encapsulated within nanoparticles have emerged as a promising strategy to counteract immunosenescence and its associated drug resistance. These HDNP-loaded nanoparticles can selectively target and eliminate senescent cells within the TME, thereby reducing SASP-mediated pro-survival signals that contribute to therapeutic resistance. Quercetin-loaded nanoparticles have demonstrated the ability to inhibit the NF-κB signaling pathway, leading to decreased secretion of IL-6 and IL-8—cytokines implicated in cancer cell survival and chemoresistance (75). Similarly, fisetin modulates the mTOR pathway and activates SIRT1, significantly diminishing SASP secretion and associated pro-survival signaling (39). Encapsulating these HDNPs within nanoparticles enhances their bioavailability and delivery precision, specifically concentrating their effects in the TME, leading to a more profound reduction in pro-survival signals and effectively reversing drug resistance.

**Figure 4:**
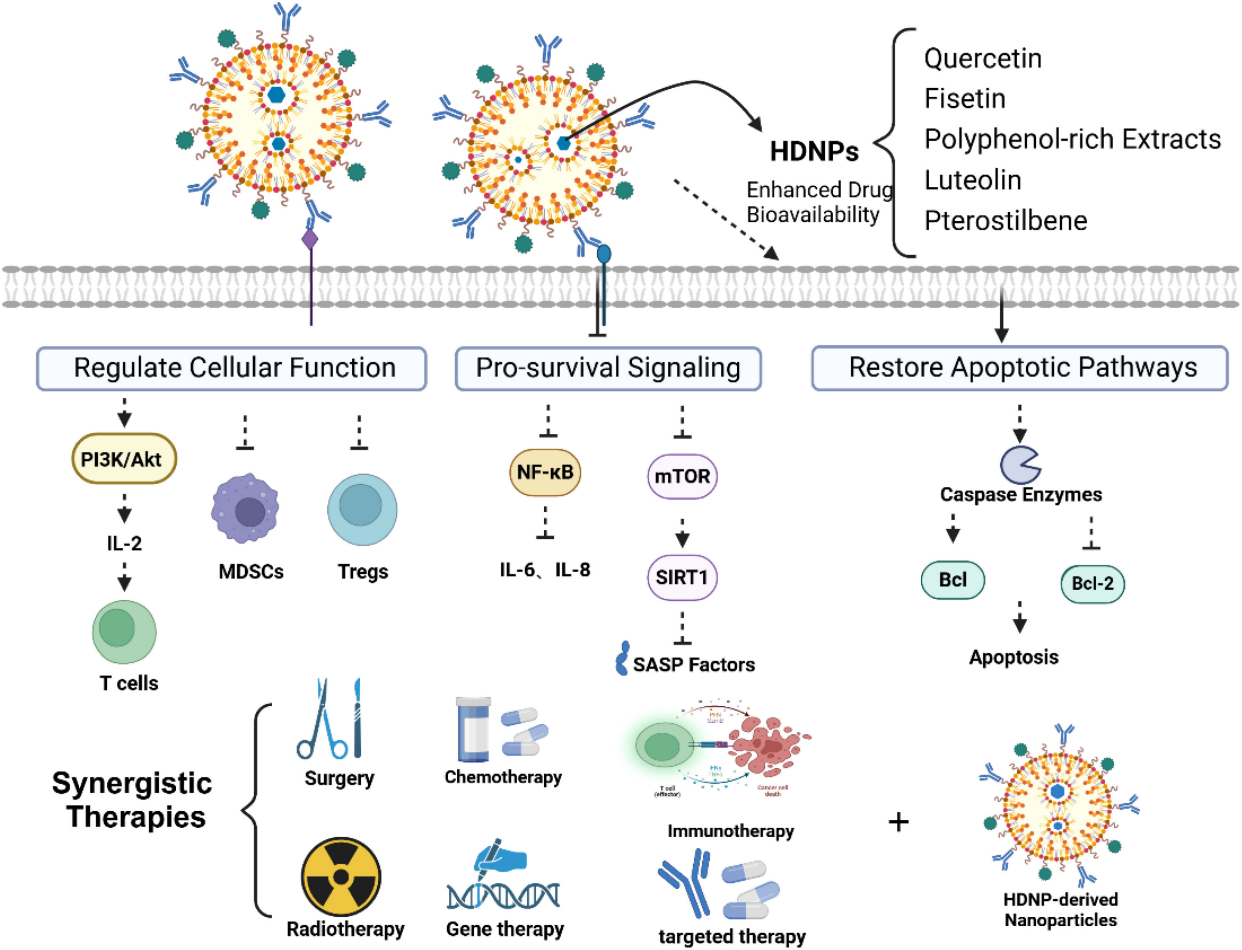
Overcoming drug resistance through modulation of immunosenescence.

Additionally, SASP factors directly inhibit apoptosis and enhance cell survival through various signaling cascades. HDNP-loaded nanoparticles can restore apoptotic signaling pathways in cancer cells compromised by SASP-driven resistance. EGCG delivered via nanoparticles has shown enhanced intracellular uptake, significantly activating apoptotic mechanisms by promoting caspase activation, upregulating pro-apoptotic proteins (e.g., Bax), and downregulating anti-apoptotic proteins (e.g., Bcl-2) (76). By overcoming bioavailability limitations, nanoparticle delivery ensures therapeutic concentrations within cancer cells, countering the anti-apoptotic effects mediated by SASP factors. (**Figure 4**).

### 5.2 Rejuvenation of immune cell function and reversal of immunosuppression

The TME characterized by immunosenescence exhibits diminished CTL functionality alongside increased populations of immunosuppressive cells such as MDSCs and Tregs, leading to compromised anti-tumor immunity (77). HDNP-loaded nanoparticles represent an innovative therapeutic strategy with substantial potential for reversing these immune deficits associated with cellular senescence, thus rejuvenating immune cell function (**Figure 4**).

Fisetin-loaded nanoparticles demonstrate notable efficacy in revitalizing senescent CTLs. By encapsulating fisetin within targeted nanoparticles, studies have shown significant improvements in the proliferation, cytotoxicity, and effector functions of CTLs, reversing signs of immunosenescence through modulation of key signaling pathways, including PI3K/Akt, and by enhancing IL-2 secretion (78, 79). These nanoparticles ensure precise delivery to aged, dysfunctional CTLs, restoring their capacity to effectively identify and eliminate tumor cells, thereby strengthening immune surveillance compromised by aging.

Quercetin-loaded nanoparticles also play a critical role in targeting immunosenescent, immunosuppressive cell populations within the TME. Research demonstrates that these nanoparticles effectively reduce both the expansion and suppressive activities of senescent MDSCs and Tregs by inhibiting immunosuppressive signaling pathways such as STAT3 and NF-κB (80). By selectively delivering quercetin to senescent immunosuppressive cells, nanoparticles enhance treatment specificity, significantly mitigating off-target effects and improving therapeutic outcomes.

Additionally, curcumin-loaded nanoparticles have been extensively investigated for their potential in reversing immunosenescence-associated immune suppression. Curcumin nanoparticles effectively reduce senescence-driven accumulation and functionality of Tregs and MDSCs, decrease levels of pro-inflammatory SASP cytokines such as IL-6 and TGF-β, and simultaneously rejuvenate effector T cell responses by modulating the inflammatory cytokine profile within the aged TME (81, 82). Further studies highlight the promising role of resveratrol-loaded nanoparticles in counteracting immunosenescence-associated immunosuppression. These nanoparticles notably reduce the recruitment and function of aged MDSCs, simultaneously promoting CTL infiltration and activation through mechanisms involving SIRT1 activation and inhibition of NF-κB signaling pathways, which are commonly dysregulated in senescent immune cells (83, 84). EGCG-loaded nanoparticles derived from green tea exhibit remarkable efficacy in overcoming immune senescence. They modulate the aged immune environment by reducing immunosenescence-induced immunosuppressive factors and promoting T cell-mediated immunity, thereby restoring a favorable milieu for immune cell activation and tumor suppression (85, 86).

Moreover, integrating multiple HDNP-loaded nanoparticles into combinational therapies represents a promising strategy to reverse comprehensive immunosenescence within the TME. Nanoparticles co-loaded with HDNPs and immune checkpoint inhibitors demonstrate enhanced therapeutic efficacy by simultaneously reversing immunosenescence-induced suppression and rejuvenating overall immune function. Nanoparticles co-delivering curcumin and anti-PD-1 antibodies exhibit synergistic effects in preclinical models, significantly improving T cell infiltration and reversing tumor-associated immune aging and dysfunction (87). Collectively, these nanoparticle-mediated HDNP strategies underscore the significant therapeutic potential in rejuvenating senescent immune cell functionality, reversing age-associated immunosuppression, and effectively overcoming immunosenescence-driven drug resistance in cancer therapy, highlighting their importance for further clinical translation.

### 5.3 Synergistic integration of HDNP-loaded nanoparticles with conventional therapies

Integrating HDNP-loaded nanoparticles with standard cancer treatments such as chemotherapy, radiotherapy, and immunotherapy represents a powerful strategy to counteract immunosenescence-driven drug resistance. These nanoparticles can potentiate conventional therapies by reducing immunosuppressive signaling, enhancing apoptosis in cancer cells, and restoring immune surveillance (**Figure 4**). The co-delivery of quercetin and doxorubicin via nanoparticles has resulted in notable synergistic effects. Quercetin inhibits drug efflux mechanisms and survival pathways, thereby significantly enhancing doxorubicin’s cytotoxicity even in resistant cancer cells (88).

Furthermore, combining HDNP-loaded nanoparticles with conventional chemotherapeutics enables dose reductions, minimizing adverse effects without compromising treatment efficacy. Studies involving curcumin nanoparticles combined with paclitaxel showed enhanced anti-tumor efficacy at lower paclitaxel doses compared to higher doses of paclitaxel alone, demonstrating the potential of nanoparticle delivery systems in enhancing therapeutic efficiency and safety profiles (89). Future research should prioritize the development of multifunctional, personalized HDNP nanoparticle systems capable of addressing multiple immunosenescence mechanisms simultaneously, optimizing clinical outcomes in cancer treatment.

## 6 Preclinical evidence and case studies

### 6.1 In vitro

Several *in vitro* studies have demonstrated that nanoparticle-mediated delivery of HDNPs effectively reduces senescence markers in cancer cell cultures. Quercetin-loaded nanoparticles have decreased the expression of senescence-associated markers in senescent tumor cells. In a study by Lewinska et al., quercetin encapsulated in nanoparticles significantly reduced senescence markers in human fibroblast cultures compared to free quercetin, indicating enhanced senolytic activity (90). Similarly, fisetin-loaded nanoparticles have been reported to suppress senescence markers in prostate cancer cells. Researchers formulated fisetin nanoparticles using PLGA (poly(lactic-co-glycolic acid)) and demonstrated that the nanoparticles reduced the expression of SASP factors such as IL-6 and IL-8 more effectively than fisetin alone (91). This suggests that nanoparticle delivery enhances the ability of HDNPs to modulate immunosenescence at the cellular level.

Nanoparticle-HDNP formulations have exhibited enhanced cytotoxic effects against drug-resistant cancer cell lines. EGCG-loaded nanoparticles were tested against multidrug-resistant breast cancer cells. Research showed that the nanoparticles increased intracellular accumulation of EGCG, leading to higher apoptosis rates in resistant cells than in free EGCG (92). The nanoparticles overcame efflux pump-mediated drug resistance by facilitating EGCG entry into cancer cells. Additionally, quercetin nanoparticles have been found to overcome resistance in ovarian cancer cells resistant to paclitaxel. Wang et al. demonstrated that quercetin nanoparticles inhibited cell proliferation and induced apoptosis more effectively than free quercetin, suggesting that nanoparticle delivery can enhance the cytotoxicity of HDNPs against resistant cancer cells (93). The study highlighted the potential of nanoparticles to sensitize resistant cancer cells to conventional therapies.

### 6.2 In vivo

*In vivo*, studies have provided evidence that nanoparticle-mediated delivery of HDNPs can delay tumor growth and reduce drug resistance. Studies investigated the effects of quercetin-loaded nanoparticles in mice bearing drug-resistant lung tumors. The treatment resulted in significant tumor growth inhibition and decreased expression of drug resistance proteins compared to controls (94). The nanoparticles improved the bioavailability of quercetin and facilitated its accumulation in tumor tissues. Similarly, Gera et al. have developed a nano-photo composite consisting of phytochemical extract (BRM270) and estimated its cytotoxic potential against HepG2 human hepatoma cancer cells. The toxicity potential of nano-photo composite against HepG2 cells was higher than free BRM270. The nano-photo composite also shows a reduction in cell growth compared to doxorubicin as it reduced concentration to 12 μg/mL. The BRM270 extract was found to downregulate specific proteins that are overexpressed during chemotherapy in HepG2 cells. The BRM270 inhibits the regulation of MMP9, BCL2, and IL 6, which are related to the potential induced apoptosis and cell proliferation (95). The enhanced anti-tumor activity was attributed to improved delivery and sustained release of drugs at the tumor site.

It is necessary to note that enhanced infiltration of functional immune cells into the tumor site is essential. Nanoparticle-HDNP formulations have been shown to modulate the tumor immune microenvironment. Treating EGCG-loaded nanoparticles in a mouse model of colon cancer led to increased infiltration of CD8^+^ cytotoxic T lymphocytes and decreased numbers of immunosuppressive MDSCs in tumor tissues (96). This shift enhanced anti-tumor immunity and contributed to tumor regression. Moreover, a study found quercetin nanoparticles to enhance NK cell activity within tumors (97). The nanoparticles improved the cytotoxic function of NK cells, contributing to tumor regression. These findings suggest nanoparticle-mediated HDNP delivery can rejuvenate immune cell functions suppressed by immunosenescence.

### 6.3 Comparative analysis

Comparative studies have consistently shown nanoparticle-mediated HDNP delivery is more effective than HDNPs administered alone. In the study, mice treated with quercetin nanoparticles exhibited more excellent tumor suppression than those treated with free quercetin, highlighting the enhanced efficacy of nanoparticle delivery (98). The nanoparticles improved pharmacokinetics and facilitated targeted delivery to tumors. Similarly, another research demonstrated that fisetin nanoparticles had superior anti-tumor effects than free fisetin in melanoma-bearing mice (99). The enhanced efficacy was attributed to increased bioavailability, targeted delivery, and sustained release provided by the nanoparticle system.

Preclinical models have shown that nanoparticle-HDNP treatments can improve survival rates and quality of life indicators compared to the HDNP monomer groups. In the study by Granja et al., mice treated with EGCG nanoparticles had prolonged survival and better physical condition than controls, indicating improved quality of life (92). The treatment was associated with reduced tumor burden and minimal toxicity. Furthermore, the study by Park et al. reported that quercetin nanoparticle treatment not only extended survival but also reduced systemic toxicity, as evidenced by stable body weight and normal organ histology in treated animals (100). These findings suggest nanoparticle-mediated HDNP delivery can enhance therapeutic efficacy while minimizing adverse effects, potentially translating into better clinical outcomes.

## 7 Clinical translation prospects and limitations

While nanoparticle-mediated delivery of HDNPs offers substantial therapeutic potential, several critical limitations and challenges must be thoroughly addressed to ensure safe and effective clinical translation. One significant challenge is ensuring the safety, biocompatibility, and controlled biodistribution of nanoparticle systems. Nanoparticles can interact unpredictably with biological systems, leading to potential toxicity, immunogenic reactions, and unintended distribution to non-target tissues (101). The physicochemical properties of nanoparticles, such as size, shape, surface charge, and composition, can influence their interaction with cells and tissues. For instance, smaller nanoparticles may penetrate tissues more deeply but pose a higher risk of crossing biological barriers and accumulating in non-target organs (101). Moreover, the long-term effects of nanoparticles in the body are not fully understood. Some materials used in nanoparticle formulations may be non-biodegradable or degrade into toxic byproducts. Thus, rigorous long-term toxicity assessments and biodegradation studies are essential before clinical applications. Furthermore, challenges in reproducibility and consistency during nanoparticle manufacturing and scaling up production processes pose significant barriers to clinical translation. The complexity of manufacturing consistent nanoparticle batches with precise physicochemical properties can complicate regulatory approval and clinical deployment.

The regulatory landscape for combination therapies that involve natural products and advanced drug delivery systems is complex. Herbal-derived compounds are often classified as dietary supplements or herbal medicines with different regulatory requirements than conventional pharmaceuticals. When combined with nanoparticles, HDNPs may fall under the category of combination products, necessitating compliance with regulations for drugs and medical devices (102). Regulatory agencies, such as the U.S. Food and Drug Administration (FDA) and the European Medicines Agency (EMA), require rigorous documentation of safety, efficacy, and quality for approval (103). Challenges include the standardization of natural product extracts, batch-to-batch consistency, and the complexity of nanoparticle characterization. Additionally, the lack of clear guidelines for nanoparticle-based natural product therapies can impede regulatory approval (21). Collaborative efforts between regulatory bodies, researchers, and industry stakeholders are needed to develop comprehensive frameworks that facilitate the clinical translation of these innovative therapies.

Although several clinical trials have explored HDNPs and nanoparticle systems in cancer treatment, clinical studies specifically targeting immunosenescence-induced drug resistance remain limited. For example, a Phase I clinical trial evaluated the safety and pharmacokinetics of nano-encapsulated curcumin (a polyphenol derived from turmeric) in patients with advanced pancreatic cancer, demonstrating acceptable safety profiles and some therapeutic benefits (104). Another trial studied the efficacy of green tea polyphenols in prostate cancer patients, showing reduced levels of prostate-specific antigen (PSA) and indicating potential anti-cancer activity (105). Advances in understanding the role of immunosenescence in cancer progression and drug resistance provide a rationale for designing clinical trials that assess the efficacy of nanoparticle-HDNP systems in modulating immune aging and improving therapeutic outcomes. In a recent study, researchers developed a self-assembling nanoparticle using the natural anticancer agent honokiol (HK) (Phase I trial CTR20170822), achieving 100% drug loading and enhanced tumor targeting via the EPR effect while improving p53-selective antitumor immunity with excellent stability and minimal toxicity (106, 107). Nonetheless, focused clinical trials assessing the efficacy and safety of nanoparticle-HDNP systems in modulating immunosenescence and overcoming drug resistance are critically needed.

Advancing clinical research should prioritize studies that explicitly target aging-related immune dysfunction. Clinical trials should emphasize elderly patient populations who are more susceptible to immunosenescence-driven drug resistance. Future research could explore combinational therapeutic approaches by integrating nanoparticle-HDNP systems with established chemotherapies or immunotherapies to potentiate clinical outcomes and reduce adverse effects. Moreover, incorporating validated biomarkers of immunosenescence, such as senescence-associated genes and immune cell profiles, could facilitate personalized treatment approaches and precise monitoring of therapeutic responses. Stratifying patients based on their immunosenescence status may further optimize therapeutic efficacy, providing essential insights into resistance mechanisms and enabling more effective patient-specific therapeutic strategies.

## 8 Conclusions

This article delves into the complex interplay between immunosenescence and drug resistance in cancer treatment. The immune aging process, characterized by altered cytokine profiles and weakened immune surveillance, plays a critical role in the emergence of drug resistance. HDNPs have shown promise in addressing immunosenescence by triggering apoptosis in senescent cells, reducing the secretion of SASP factors, and boosting the functionality of immune cells. Enhancing the stability, solubility, and targeted delivery of HDNPs to the TME and senescent immune cells via nanoparticles has amplified their therapeutic potential. These advancements have demonstrated an ability to decrease senescence markers, improve cytotoxic effects on resistant cancer cell lines, delay tumor progression, and increase the infiltration of functional immune cells into tumors. Although preclinical studies show great potential, more research is necessary to comprehensively understand the mechanisms through which immunosenescence promotes drug resistance and how HDNP-loaded nanoparticles can modulate these pathways. Further investigation into additional senolytic HDNPs and the refinement of nanoparticle formulations could lead to more potent therapeutic options. In conclusion, nanoparticle-mediated HDNP delivery offers an innovative and promising approach to overcoming drug resistance caused by immunosenescence in cancer therapy. By addressing the core mechanisms of immune aging, this strategy can potentially improve treatment efficacy and enhance the quality of life for cancer patients.
